# Community Health Worker Program Outcomes for Diabetes and Hypertension Control in West Bank Refugee Camps: A Retrospective Matched Cohort Study

**DOI:** 10.9745/GHSP-D-22-00168

**Published:** 2022-10-31

**Authors:** Asmaa Rimawi, Adarsh Shah, Henry Louis, David Scales, Jawad Abu Kheiran, Nashat Jawabreh, Sofia Yunez, Masako Horino, Akihiro Seita, Bram Wispelwey

**Affiliations:** a Harvard T.H. Chan School of Public Health; Harvard Medical School, Boston, MA, USA.; b Harvard Kennedy School, Harvard University, Cambridge, MA, USA.; c University of Massachusetts Chan Medical School, Worcester, MA, USA.; d New York-Presbyterian Hospital, Weill Cornell Medicine, New York, NY, USA.; e United Nations Relief and Works Agency, Arroub refugee camp, occupied Palestinian territory.; f Makassad Hospital, East Jerusalem, occupied Palestinian territory.; g University of Illinois College of Medicine, Chicago, IL, USA.; h United Nations Relief and Works Agency, Amman, Jordan.; i Brigham and Women’s Hospital, Harvard Medical School; Harvard T.H. Chan School of Public Health, Boston, MA, USA.

## Abstract

**Introduction:**

Community health worker (CHW) programs have proven effective in improving diabetes control in many locations and settings, but data on feasibility and efficacy are lacking in the Middle East and settings of chronic violence. A Palestinian CHW program, Health for Palestine (H4P), addresses chronic diseases in West Bank refugee camps. Our study assesses the feasibility and effectiveness of the program’s diabetes and hypertension interventions.

**Methods:**

Data on home visits, patient retention, and blood pressure were extracted from the CHW records and analyzed. To assess diabetic patient progress, we conducted a retrospective matched cohort study using data obtained from a United Nations (UN) clinical database to analyze the trajectory of hemoglobin A1c (A1c) values. Thirty of the 47 diabetic patients in the H4P CHW program met study inclusion criteria and were each matched with 3 patients from the Bethlehem UN clinic (n=120). We tested for significance using multivariable linear regression with robust standard errors.

**Results:**

The average number of home visits per patient per month was 7.3 (standard deviation=4.1), and the patient retention rate was 100% over an average of 11.2 months. For hypertension patients in the CHW program (n=33), mean systolic blood pressure decreased by 7.3 mmHg (95% confidence interval [CI]=1.93, 12.25; *P*=.009) and mean diastolic blood pressure by 4.3 mmHg (95% CI=0.80, 7.91; *P*=.018) from March 2018 to November 2019. On average, diabetic patients within the CHW group experienced a 1.4 point greater decline in A1c per year compared to those in the non-CHW group, after adjusting for potential confounders (95% CI=−0.66, −2.1; *P*<.001).

**Discussion:**

The results suggest that CHW accompaniment may be an effective model for improving diabetes and hypertension control in refugee camps experiencing direct violence and extreme adversity. A low exclusion cut-off for A1c (≤6.4%) may underestimate the program’s impact.

## Abstract (precis)

A community health worker program in urban Palestinian West Bank refugee camps improves diabetes and hypertension control in a setting of chronic violence and extreme adversity.

## Teaser

**Key Findings:**

- Health for Palestine (H4P) was established in 2018 as a refugee-run community health worker program in the occupied West Bank to provide social accompaniment, address health barriers, and initiate monitoring and support for chronic disease patients.
- A study from 2 Palestinian refugee camps indicates that accompaniment-based community health worker programs are feasible and effective in improving diabetes and hypertension control in camps experiencing chronic violence under military occupation.

**Key Implications:**

- Stakeholders providing health care to refugees in Palestine and the diaspora can consider expanding an intensive community-run health model to other camps and communities experiencing extreme adversity as a result of occupation, dispossession, and violence.
- Given the rapidly increasing rise in migration globally, community health programs like H4P could play a key role in mitigating health barriers and maximizing community agency in similar contexts of extreme adversity. Policy makers should consider the multimodal benefits of integrating such programs into broader health programming.

## INTRODUCTION

Among adult refugees in the West Bank, the prevalence of type 2 diabetes mellitus (T2DM) rose to a striking 14.8% in 2017, significantly higher than the global prevalence (8.8% in 2017).^1^^,^^2^ In 2018, this prevalence increased to 17.6%,^3^ and this is likely an underestimate.^4^ The majority (74.4%) of T2DM patients in West Bank refugee camps have disease that is uncontrolled,^5^ placing them at high risk for the numerous associated complications, including blindness, kidney failure, heart attack, and lower limb amputations.^6^ The United Nations Relief and Works Agency for Palestine Refugees in the Near East (UNRWA), the primary health care provider for Palestinian refugees in the West Bank, has offered health care services for diabetic patients since 1992. Funding cuts by the United States government in 2018 to UNRWA left the agency US$1.2 billion in debt, resulting in cutbacks in crucial health care services, including diabetes care for Palestinians.^7^

Community-based treatment approaches are responsible for some of the most exciting advancements in global health delivery in recent years.^8^^–^^10^ Community health worker (CHW) programs have proven effective in improving diabetes control in the urban United States^11^^–^^13^ and other low- and middle-income countries,^14^^–^^18^ but data in the Middle East and North Africa region are lacking. A handful of studies have assessed the impact of CHW programs among refugee communities,^19^^–^^24^ but there are only a few studies on CHW programs within refugee camps^25^^,^^26^ and even fewer within zones of active violence.^27^ An informal review revealed only 1 major study on CHW programs in Palestine^28^ and no major studies on CHW programs in Palestinian refugee camps. The Ghosh et al. study, completed in 2007, assessed multiple medical interventions conducted for diabetic patients in 3 West Bank villages, among them a CHW program. The specific role of CHWs within this intervention is not clarified, and no *P* value was listed for the 0.75 point decline in hemoglobin A1c (A1c) reported.

### Program Model

In early 2018, a home-based CHW program comprised entirely of Palestinian refugees, Health for Palestine (H4P), was initiated as a joint program between 1for3, a Boston-based nongovernmental organization (NGO) serving Palestinian communities globally, and the Lajee Center, a community-based grassroots cultural center in Aida and Beit Jibrin (also commonly known as Al-Azza) camps in Bethlehem, the occupied West Bank. Aida and Beit Jibrin refugee camps, with populations of 5,500 and 2,900, respectively, comprise refugees from more than 27 villages in what is now Israel. The population density of these camps is more than twice that of Manila, the most crowded city in the world.^29^

The program was created to address the top health concerns of Aida and Beit Jibrin camps’ residents and their most common chronic diseases, including hypertension and T2DM.^30^ The initial CHWs, all 6 of whom are refugees from the camps aged 19–26 years at the time of the study, were recruited by the Lajee Center using posted announcements in the camps and on social media. An internal medicine physician and a medical student trained the CHWs using a guide modified from published CHW manuals. The training period lasted 6 weeks and included lectures, role playing, hands-on training for blood pressure and blood glucose measurement, and ultimately guided home visits with a physician and/or nurse.

H4P was created to address the top health concerns, including hypertension and T2DM, of Aida and Beit Jibrin camps’ residents.

Initial remuneration of the CHWs was 1500 New Israeli Shekels (NIS) (∼US$428) per month in 2018, which, although the minimum wage in the occupied West Bank, is a coveted salary given high rates of unemployment particularly among youth and in refugee camps.^31^ Salaries were increased to NIS1800 (∼US$514) per month in 2019, and 4 additional CHWs were hired that year at the starting salary. CHWs coordinate closely with a pharmacist and physician to adjust patient care plans and to accompany patients to clinics and hospitals as needed, and the CHWs are supervised by a project manager who is also a licensed nurse. A backup medicine supply is maintained to mitigate frequent local stock-outs at both UNRWA and Palestinian Ministry of Health pharmacies. Continuing education is delivered by volunteer physicians and medical students from the United States and Palestine, and the program is financially supported by family foundations and individual donations to 1for3 and the Lajee Center. While no formal costing analysis has yet been published, and despite a more intensive longitudinal focus serving fewer patients per CHW, H4P costs are in the vicinity of an established CHW program in Nepal that includes a noncommunicable disease component.^32^ H4P’s per capita annual cost of US$6.49 (versus US$3.05), 85% of which is personnel costs (versus 74%), and a CHW-to-population ratio of 1:1400 (versus 1:2017) are comparable, particularly as H4P’s newly trained CHWs had not yet maximized their patient panels (i.e., peak efficiency) during this study’s time window. Furthermore, compared with the 2011 UNRWA cost per visit-minute of US$5.99 in the West Bank,^33^ H4P’s cost per visit-minute is more than 16 times lower at US$0.36.

The H4P CHW intervention was designed to mitigate the fragmentation of care resulting from settler colonialism and the complexities of a prolonged refugee crisis produced by the *Nakba* (“catastrophe”) of 1948, in which 85% of Palestinians were displaced, fled, or were massacred.^30^^,^^34^ The impacts of a fragmented health system, intergenerational trauma of the Nakba, and ongoing military occupation of the West Bank have resulted in devastating health effects for Palestinians, and particularly refugees within overcrowded refugee camps.^35^ Poorly connected networks of health care provided by public, private, NGO, charity, and multilateral actors, as well as logistical dilemmas generated by military checkpoints and the colonial architecture of occupation,^36^ have resulted in a health care system that is difficult to access and navigate. H4P’s work is grounded in the goal of promoting enhanced community control over health, easing patient navigation of these fragmented networks, and simplifying and resisting the resulting complexities in accessing basic, effective, and longitudinal care for refugees living in camps.

H4P’s work is grounded in the goal of promoting enhanced community control over health.

The CHW intervention takes an accompaniment approach,^37^ which encompasses transportation facilitation, medication supervision and delivery, adherence support, appointment scheduling and adherence, and monitoring of vital signs and glucose. The CHWs utilize motivational interviewing to address the primary risk factors of diet, exercise, medication adherence, and smoking. Given the known impacts of stress and allostatic load on chronic diseases like diabetes,^38^ psychosocial counseling is integrated into CHW patient care. For guidance and support, the CHWs meet weekly with a social worker, also a Palestinian refugee, who is supervised by a clinical psychologist. The social suffering from occupation, military incursions, and prolonged displacement influence a range of psychological and physiological outcomes that interact to heighten morbidity as described in syndemic models.^39^^–^^41^ Understanding the efficacy of H4P’s work among Aida and Beit Jibrin camp residents, a population suffering regular military incursions and the greatest exposure to tear gas in the world,^42^ may offer new insight into the role that CHW programs can play within the Middle East, refugee camps, and zones of chronic violence and social exclusion or oppression.

**Figure:**
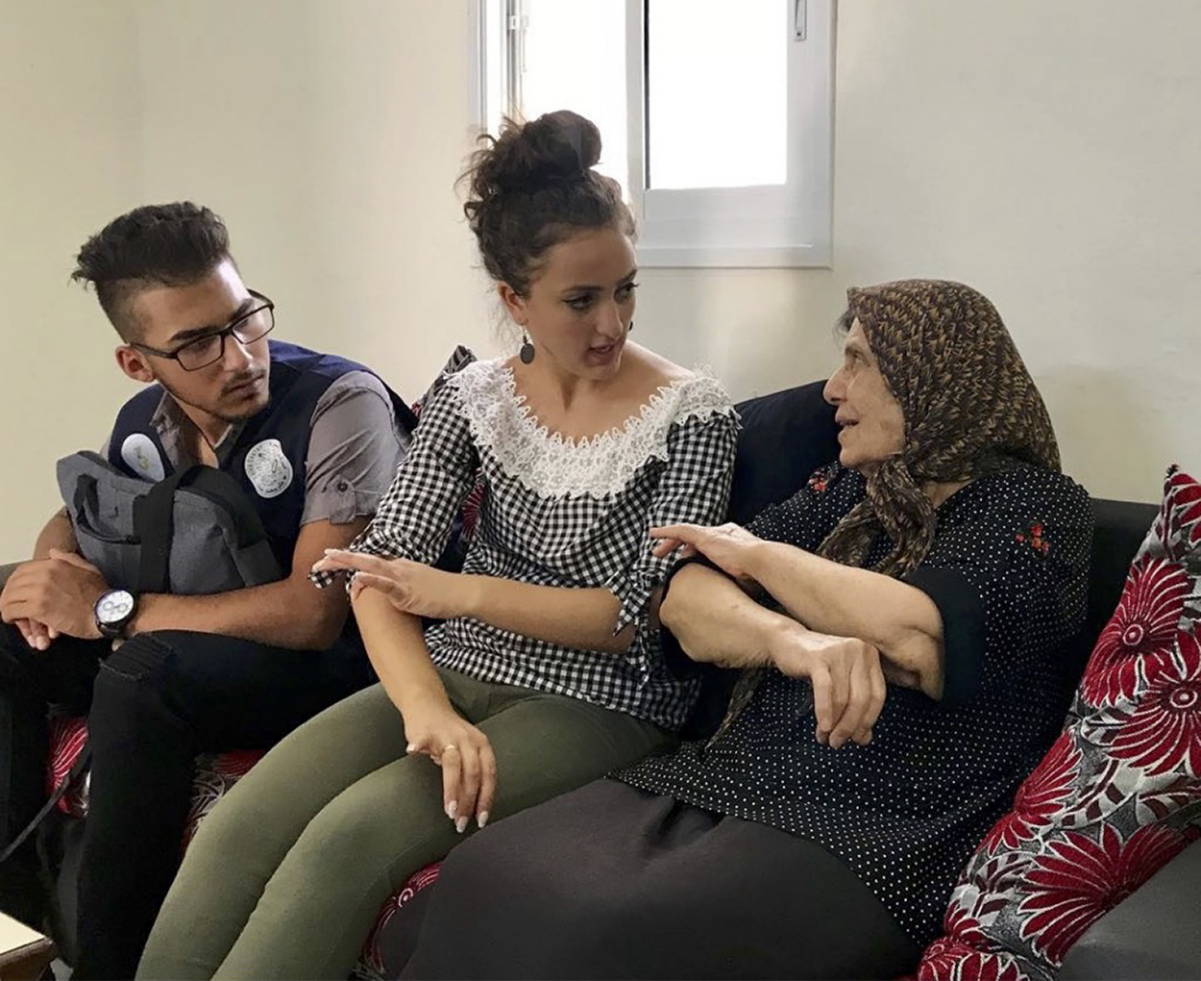
Community health workers in the West Bank discuss symptoms with a patient. © 2018 Nashat Jawabreh/Health for Palestine

As 1 of the first studies to assess an accompaniment-based CHW program in the region, this study aimed to (1) understand the feasibility of a grassroots accompaniment-based CHW program in a high-conflict and limited-resource setting, (2) assess the mean trend in patient blood pressure in hypertensive patients over the course of the first year of the program, and (3) assess the association between being assigned an H4P CHW as a T2DM patient in Aida or Beit Jibrin camp on A1c change. Our hypothesis was that CHW assignment results in greater diabetes progress as indicated by a declining A1c value compared to patients without CHW assignment.

## METHODS

### Study Population

At the time of the initiation of the data analysis in late 2019, H4P had cared for 101 patients since its founding in 2018. We recruited patients living within Aida and Beit Jibrin camps into the program using several techniques, including flyers, personal connections to hired CHWs, and most commonly, snowballing. Recruitment targeted no specific centers, geographic areas, or patient types. A simple visual geospatial analysis of a random sample of patients revealed no grouping of patients by location, such as in the camp center, near medical clinics, or around the CHWs’ office at Lajee Center.

To enroll in the CHW program, patients had to have a known diagnosis of hypertension or diabetes, be aged over 18 years, and reside in Aida or Beit Jibrin camp. We confirmed a diagnosis of diabetes or hypertension using a medical history intake form compiled from clinic notes and patient interviews. Study eligibility criteria for diabetes patients included receiving diabetic care solely within the UNRWA Bethlehem clinic, a baseline A1c greater than 6.4, at least 2 A1cs at least 6 months apart during the duration of the program, and 1 A1c no earlier than 5 months before the first visit recorded by the CHWs. For hypertension patients, eligibility criteria for inclusion in the blood pressure analysis were (1) a diagnosis of hypertension, (2) blood pressure values available 9–12 months after enrollment, and (3) baseline blood pressure greater than or equal to 130/80.

Matches were selected from among all diabetic patients receiving diabetic care at the UNRWA Bethlehem clinic, with an A1c test performed between March 2018 and November 2019. These dates correspond with the duration of the H4P program and were selected to ensure similar start and end dates of both groups. We extracted data on matched patients from the UNRWA electronic medical record system.

### Patient and Public Involvement

The concept of patient involvement is integral to the CHW intervention and extended to the goals of this analysis. Plans for the retrospective study were born out of patient interest in understanding their own progress and were shared with the 30 patients receiving CHW care. Patient interest in viewing their A1c results extracted from the UNRWA database for the purpose of the analysis resulted in a modified data agreement with UNRWA to allow transfer of patient A1c values into CHW data forms via KoBoToolbox for permanent patient access. All 30 patients receiving CHW care were given updates on both their individual A1c trajectory over time and the average A1c decline among the intervention group. The medical implications of A1c change were shared with the CHWs, who shared this information with patients on an individual level.

### Intervention

The intervention analyzed is the aggregate chronic disease program conducted by H4P’s CHWs, whose core tasks are implemented as home-based care. Home visits are prioritized according to 3 levels of disease control, with the patients with the most controlled parameters being visited at least twice a month and those with the most uncontrolled parameters up to multiple times per week. Patients are graduated from the CHW program once they have spent 6 consecutive months at the highest level of control. At its foundation, each visit includes an assessment of the patient’s (1) blood pressure, (2) random blood glucose level, and (3) medication supply and adherence. CHWs measure glucose with locally available fingerstick testing glucose monitors according to manufacturer instructions, and measure blood pressure using automatic instruments according to standard protocol.^10^ CHWs engage in active listening and trauma-informed discussion of health challenges, with motivational interviewing and counseling on diet, exercise, smoking, and medication adherence. All patients receive a pillbox from the CHWs with instructions on its proper use. A local physician and a CHW manager closely supervise the CHWs to ensure regular visits and high-quality reporting. International advisors assist in strategic planning and assessments of data quality. The nonintervention group received the standard of care at the Bethlehem UNRWA clinic, which typically consisted of annual follow-up visits with a physician at the clinic.

**Figure:**
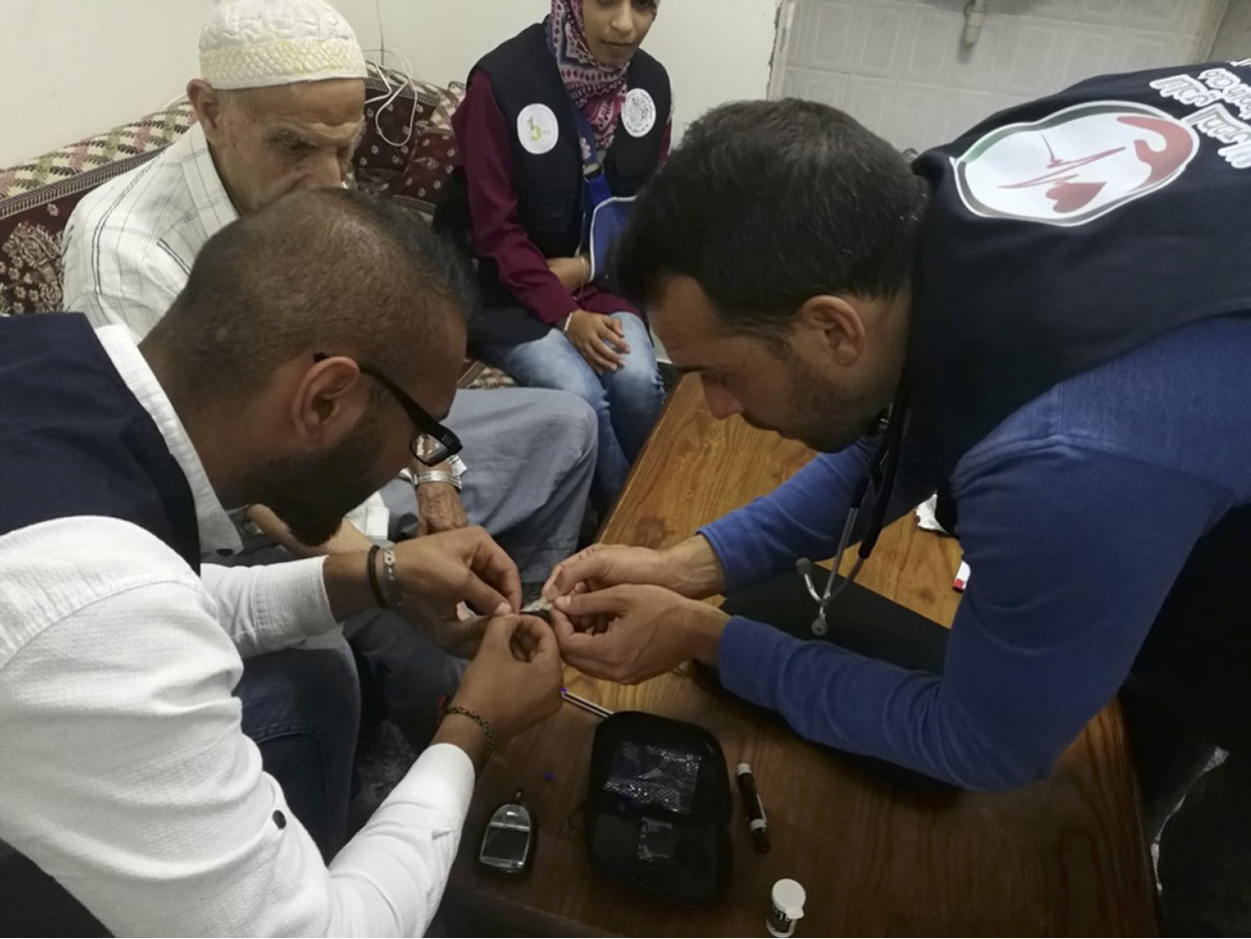
Community health workers in the West Bank check a patient’s blood glucose during one of their first home visits. © 2018 Nashat Jawabreh/Health for Palestine

CHWs engage with patients in active listening and trauma-informed discussion of health challenges.

### Study Design

Feasibility measures include visit metrics, specifically number of visits accomplished and patient acceptance of medical assistance from a CHW, including unscheduled home visits. CHWs record each visit, including its length and the metrics collected, within a customized data form on KoBoToolbox for later access and analysis. Baseline blood pressures for hypertensive patients were measured by CHWs at entry into the CHW program and at each subsequent visit using an automatic blood pressure cuff.

Assessment of early outcomes for diabetic patients included analysis of patient A1c values over the duration of their enrollment in the program, from March 2018 to November 2019. As part of the CHW program, all enrolled diabetic patients had undergone A1c testing (ranging from 1 to 4 tests) over the duration of the program’s intervention, the results of which were extracted from the UNRWA database for the purpose of this study. Timing of intervention, as well as timing of repeat measurements, varied from patient to patient. Each patient who received CHW care was retrospectively matched to 3 T2DM patients receiving diabetic care primarily at the UNRWA Bethlehem clinic, by prespecified characteristics and ranges, specifically: baseline A1c (+/− 0.2), age (+/− 3 years), gender (female/male), and follow-up time (+/− 4 months). Logical statements were used for the matching (with replacement) process. Baseline age, gender, history of hypertension, stroke, myocardial infarction, and A1c levels were collected from UNRWA records for all patients.

### Statistical Analysis

We accessed average blood pressure readings between March 2018 and November 2019 for all patients at baseline and 9–12 months post-enrollment, using the last blood pressure reading closest to 12 months, and analyzed the data using Excel. Standard deviations are reported and statistical significance for comparisons between baseline and 1 year were computed using 2-sided paired t-tests. Blood pressure data for controls was not uniformly available and so no direct comparison was made.

We created a multivariable regression model to test the hypothesis that CHW assignment results in greater diabetes progress as indicated by a declining A1c value compared to patients without CHW assignment. Unadjusted differences in means for patients in the treatment and control groups are also reported. Given the vast literature on the subject of A1c change, we used clinical reasoning to select covariates for the multivariable model. Despite the unsystematic entry of participants into the CHW program, 5 prespecified covariates were included given their role as potential confounders: history of myocardial infarction, history of stroke, physical activity status, smoking status, and hypertension status. We also performed an assessment without outliers since only 1 follow-up A1c value for each patient was available for the analysis. To account for this second analysis, we used a Bonferroni correction, with 2-tailed *P*-values of .025 considered statistically significant (.05/2). However, since the analysis was conducted for presentation of results, we did not use a Bonferroni correction for the inclusion of unadjusted results. All confidence intervals (CIs) were calculated with the use of heteroskedasticity-robust standard errors. Because our data set includes 120 subjects and we considered 6 variables in our model, overfitting was not a concern.

Due to the small number of patients within the CHW group, Fisher's exact analysis was used to compare proportions of patients under diabetic control (defined as A1c<8%). Data were analyzed using SAS version 9.4.

### Ethical Approval

Institutional review board approval was not required for this retrospective matched cohort study per the Institutional Review Board of Harvard Medical School (Protocol 19-0081), which was locally reviewed for ethical risk and approved by H4P’s partnering organization, Lajee Center, in Aida camp. All participants gave their informed verbal consent or had already done so as part of the care received by the governing organizations.

## RESULTS

### Participants

**FIGURE 1:**
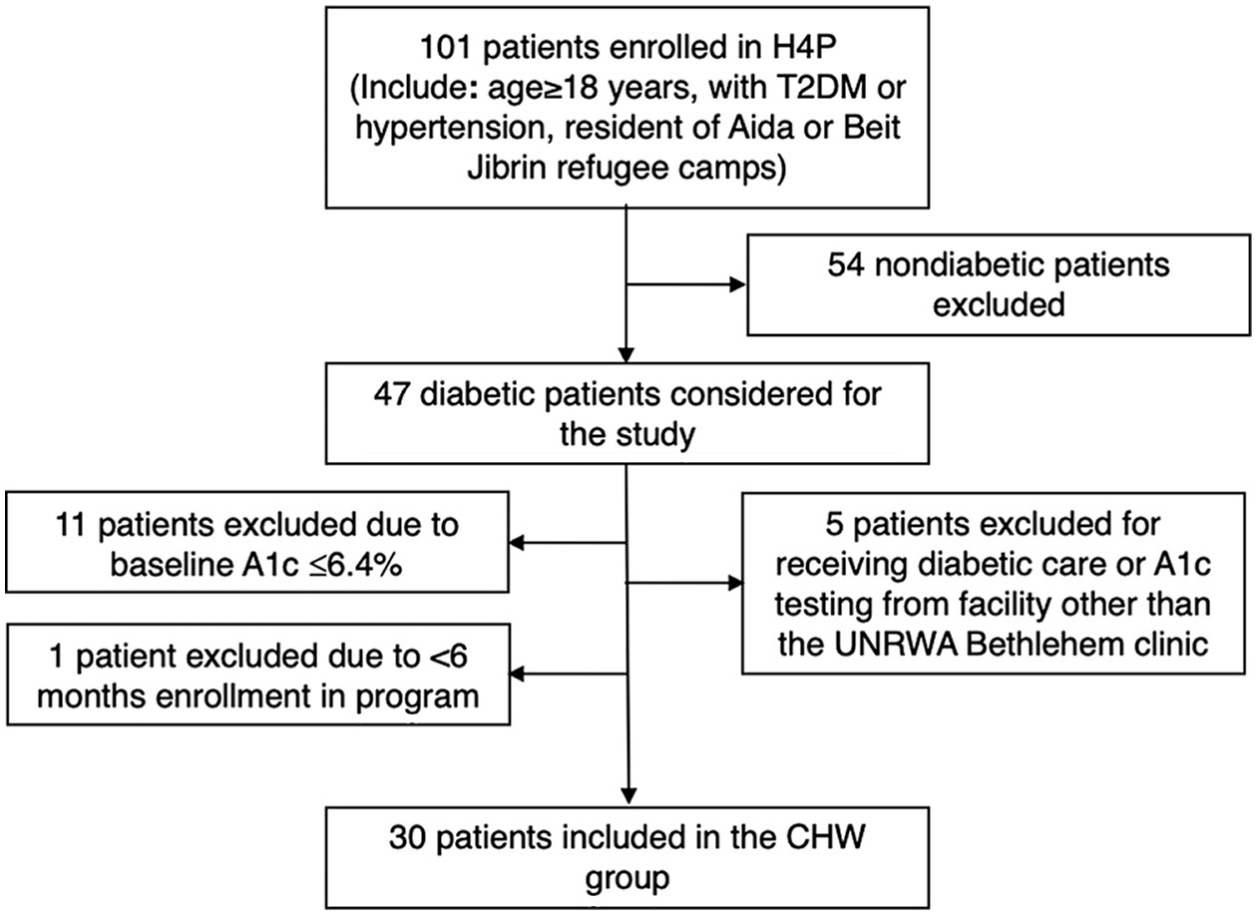
Selection of Intervention Patient Group, Bethlehem Governorate, West Bank^a^ Abbreviations: A1c, hemoglobin A1c; CHW, community health worker; H4P, Health for Palestine; T2DM, type 2 diabetes mellitus; UNRWA, United Nations Relief and Works Agency for Palestine Refugees in the Near East. ^a^ Of the 101 patients enrolled in H4P, 47 of the patients had a diagnosis of T2DM upon enrollment into the program. Of these 47 patients, 11 patients were excluded from the study for having a baseline hemoglobin A1c of less than or equal to 6.4. An additional 5 patients were excluded for receiving any diabetic care and A1c testing from a facility other than the UNRWA Bethlehem clinic. One patient was excluded for being in the program for less than 6 months. The remaining 30 patients met the eligibility criteria and were included in the study as the intervention group, or “with CHW care” group.

Of the 101 patients enrolled in H4P in November 2019, 33 were eligible for the blood pressure analysis in having (1) a diagnosis of hypertension, (2) blood pressure values available 9–12 months after enrollment, and (3) baseline blood pressure greater than or equal to 130/80. Forty-seven of the patients had a diagnosis of T2DM upon enrollment into the program, and all were considered for inclusion into the study (Figure 1). Of these 47 patients, 11 patients were excluded from the study for having a baseline A1c of less than or equal to 6.4%, meaning they did not stand to benefit from further diabetic control or may not have had a confirmed diagnosis of T2DM. An additional 5 patients were excluded for receiving any diabetic care and A1c testing from a facility other than the UNRWA Bethlehem clinic; their A1c values are reported for completion (Supplement Table S1). One patient was excluded for being in the program for less than 6 months. The remaining 30 patients met the eligibility criteria and were included in the study as the intervention group, or “with CHW care” group; their A1c values were extracted from the UNRWA electronic medical record.

**FIGURE 2:**
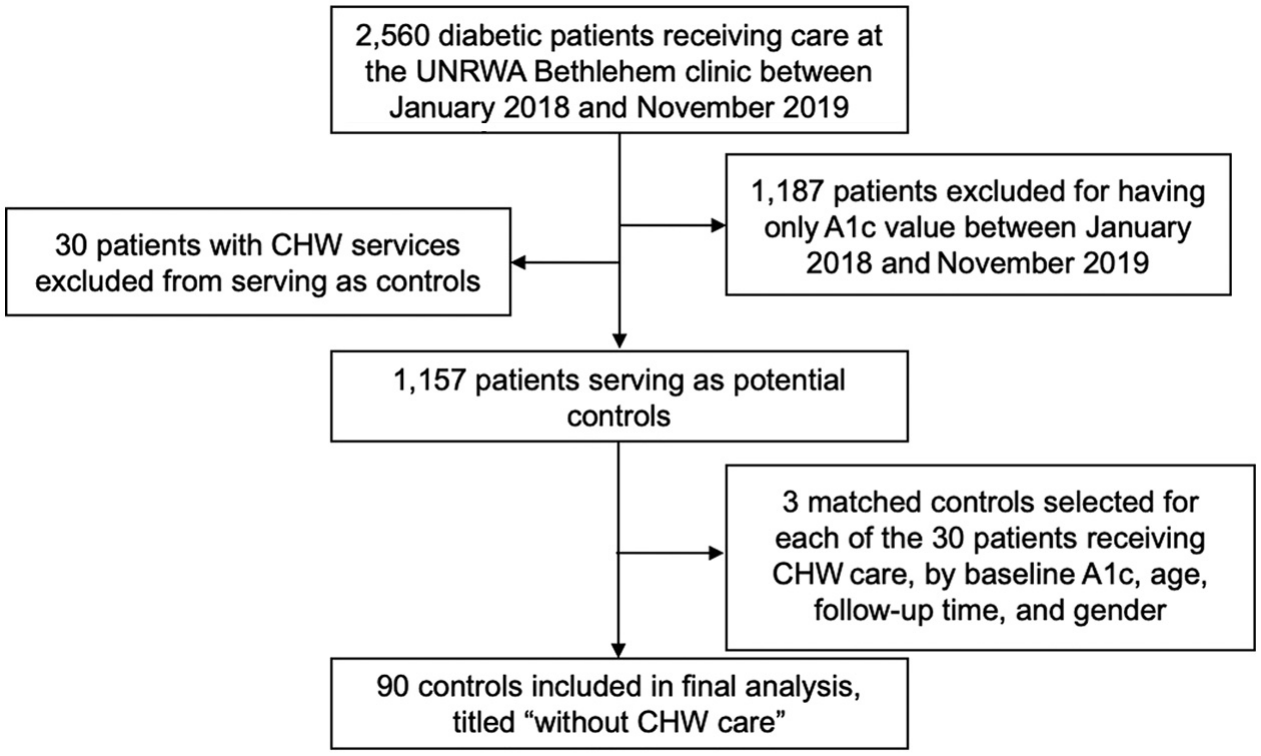
Selection of Control Patient Group, Bethlehem Governorate, West Bank^a^ Abbreviations: A1c, hemoglobin A1c; CHW, community health worker; UNRWA, United Nations Relief and Works Agency for Palestine Refugees in the Near East. ^a^ Data on potential matches included 2,374 potential controls. 1,187 patients without a second A1c value in the UNRWA records were removed from the database. Matches were selected from the remaining 1,157 potential controls and served as the “without CHW care” group. Three matches were selected for every patient within the intervention group.

Data on potential matches were provided by the same UNRWA electronic medical record and included 2,374 potential controls. Because 2 A1c values were needed for the purpose of the study, 1,187 patients without a second A1c value in the UNRWA records were unable to serve as a matched control and were removed from the database. Matches were selected from the remaining 1,157 potential controls and served as the “without CHW care” group (Figure 2). Three matches were selected for every patient within the intervention group, resulting in a study size of 120.

Baseline patient characteristics, including smoking status, physical activity status, history of stroke or myocardial infarction, and prescription of insulin, oral hypoglycemic agents, or antihypertensive medications, on both the intervention (with CHW care) and final control group (without CHW care), were also sourced from the UNRWA database (Table 1).

**TABLE 1.** Characteristics of Patients with Type 2 Diabetes, Bethlehem Governorate, West Bank^a^

**Patient Characteristics**	**With CHW Care (n=30)**	**Without CHW Care (n=90)**	***P* Value **
Mean age, years (range)	62.3 (38–84)	61.8 (39–83)	.71
Women, No. (%)	19 (63.3)	57 (63.3)	.83
Mean baseline A1c (SD)	8.6 (1.7)	8.6 (1.7)	.89
Mean follow-up time, months	11.2	12.3	.06
Prescribed insulin, No. (%)	10 (33.3)	41 (45.6)	.29
Prescribed OHA, No. (%)	30 (100)	48 (53.3)	.19
Prescribed anti-HTN, No. (%)	26 (86.7)	79 (87.7)	1.0
Physical activity, No. (%)	8 (26.7)	9 (10)	.03
Smoker, No. (%)	8 (26.7)	3 (3.3)	.001
Myocardial infarction history, No. (%)	7 (23.3)	10 (11.1)	.13
Stroke history, No. (%)	2 (6.7)	1 (1.11)	.15

Abbreviations: A1c, hemoglobin A1c; anti-HTN, antihypertensive medications; CHW, community health worker; OHA, oral hypoglycemic agent; SD, standard deviation.

^a^ T-test for age and baseline A1c, Satterthwaite for follow-up time, Fisher’s exact for binary variables.

### Feasibility

CHWs completed 1,931 patient visits across the 30 patients with CHW care. The study period lasted from March 2018 to November 2019, with an average enrollment length of 11.2 months, ranging from a minimum of 9 months enrollment length to 18 months. The average number of visits was 7.3 visits per month per patient (standard deviation [SD]=4.1), with an average of 17.2 minutes per visit (SD=9.1). The minimum number of patient visits was 23 visits per patient across an enrollment period of 9 months, with a maximum of 167 patient visits across an enrollment period of 13 months. All 30 patients (100%) were retained in care throughout the study period.

### Blood Pressure Trajectory

In patients with hypertension and baseline blood pressure greater than or equal to 130/80 mmHg, average systolic blood pressure decreased by 7.3 mmHg from 138 mmHg to 131 mmHg (95% CI=1.93, 12.25; *P*=.009), and average diastolic blood pressure decreased by 4.3 mmHg from 85 to 80 (95% CI=0.80, 7.91; *P*=.018) over 9–12 months (Table 2). After 1 year in the CHW program, 58% of patients had a blood pressure less than 130/80 (*P*<.001). For patients with baseline stage II hypertension (≥140/90, 64% of the cohort), only 18% remained at stage II 1 year later (*P*<.001).

**TABLE 2.** Hypertension Patient Outcomes, Bethlehem Governorate, West Bank^a^

	**Hypertension Patients (n=33)**			
	**Baseline**	**After 1 Year**	***P* Value**	**Mean Difference (95% CI)**
Average BP, mmHg, systolic/diastolic (SD)	138/85 (13/8)	131/80 (12/10)	.009/.018	7.3 (1.93, 12.25) / 4.3 (0.80, 7.91)
Controlled hypertension (BP<130/80), No. (%)	0 (0)	19 (58)	<.001	N/A
Stage II hypertension (BP≥ 140/90), No. (%)	21 (64)	6 (18)	<.001	N/A

Abbreviations: BP, blood pressure; CI, confidence interval; N/A, not applicable; SD, standard deviation.

^a^ Paired 2-sided t-test.

After 1 year in the CHW program, 58% of patients had a blood pressure less than 130/80.

### A1c Trajectory

The mean time between the baseline A1c measurement and the follow-up A1c measurement was 11.2 months in the CHW group, and 12.3 months in the non-CHW group, for an overall average of 12 months. Using the multivariable model with robust standard errors, we found that those within the CHW group saw a mean 1.4 point greater decline in A1c per year compared to those in the non-CHW group, after adjusting for potential confounders (95% CI=−0.66, −2.1; *P*<.001) (Table 3 and Supplement Table S2). The unadjusted reduction in A1c for patients in the CHW group was 1.2 points (95% CI=−0.51, −1.88; *P*<.001), and the unadjusted A1c change in the non-CHW group was 0.08 points (95% CI=−0.2, 0.36; *P*=.56).

**TABLE 3.** Diabetes Patient Outcomes, Bethlehem Governorate, West Bank^a^^,^^b^

**Predictor**	**Coefficient for A1c Change**	***P* Value**	**95% CI**
Unadjusted key measures			
Enrollment in CHW intervention	−1.2	<.001	−0.51, −1.88
No enrollment in CHW intervention	0.08	.56	−0.2, 0.36
Adjusted key measures from multivariable linear regression model			
Enrollment in CHW intervention	−1.4	<.001	−0.66, −2.1

Abbreviations: A1c, hemoglobin A1c; CHW, community health worker; CI, confidence interval; UNRWA, United Nations Relief and Works Agency for Palestine Refugees in the Near East.

^a^ Shown are the mean values for the outcome in the CHW group and the non-CHW group. Calculation of the unadjusted mean A1c change was based on an indicator from an ordinary least-squares regression of the outcome, with no other covariates. Calculation of the adjusted A1c change was based on an indicator for the CHW group from an ordinary least-squares regression of the outcome with prespecified covariates. All CIs were calculated with the use of heteroskedasticity-robust standard errors. Prespecified covariates included history of myocardial infarction, history of stroke, physical activity status, smoking status, and hypertension status. All covariates were measured at time of baseline A1c and were extracted from the UNRWA Bethlehem clinic medical records.

^b^ See further coefficients in Supplement Table S2.

To ensure robustness of the results, we repeated the analysis after removing 2 patients with high residuals and high leverage, with no change in clinical or statistical significance of the results. The same multivariable model with robust standard errors was used for this analysis, and the adjusted difference for A1c change among those within the CHW group compared to those in the non-intervention group was −1.1 points (95% CI=−0.55, −1.56; *P*<.001). Both outliers were from the CHW group and saw marked decline in A1c (5.3 points and 6.5 points). For discussion of the results, we included these patients with outlying A1c values in the final analysis because we have no reason to believe their A1c levels were differentially measured and their inclusion eases the interpretation of our results.

**FIGURE 3:**
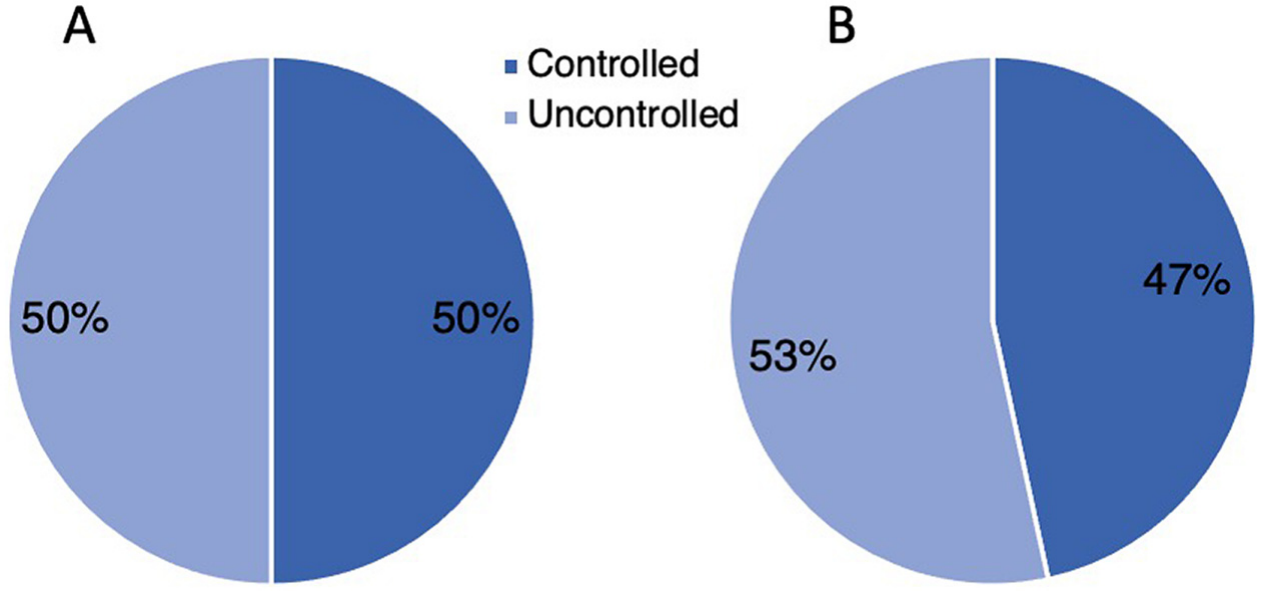
Status of Type 2 Diabetes Patients at Baseline, Bethlehem Governorate, West Bank in CHW Group (A) and Non-CHW Group (B) Abbreviation: CHW, community health worker.

**FIGURE 4:**
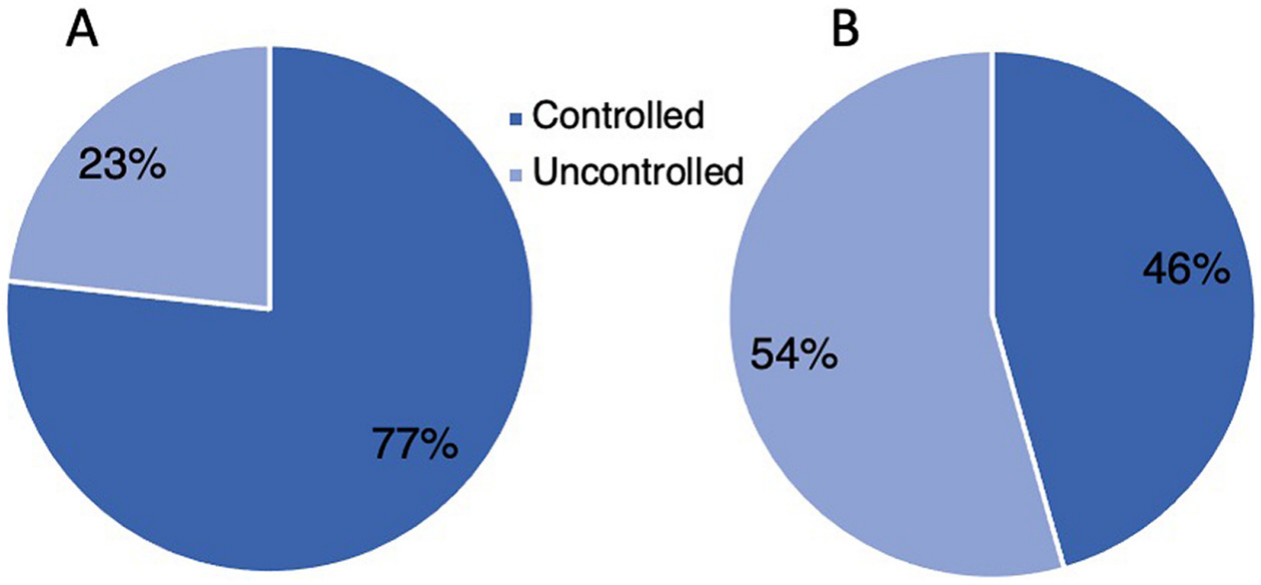
Status of Type 2 Diabetes Patients at Follow-up, Bethlehem Governorate, West Bank in CHW Group (A) and Non-CHW Group (B) Abbreviation: CHW, community health worker.

Among the 30 patients with CHW care, 50.0% of the CHW patient group compared to 46.7% of the non-CHW patient group were controlled at baseline (Fisher’s exact, *P*=.83) (Figure 3). At the end of the study, 76.7% met the glycemic target for A1c less than 8%, compared to 45.6% of the 90 patients without CHW care (Fisher’s exact, *P*=.003) (Figure 4).

## DISCUSSION

These results suggest that a CHW intervention grounded in accompaniment is a feasible and effective model for improving chronic disease control in urban refugee camps in a setting of persistent direct violence and military occupation. Despite a poverty rate of nearly 40% among Palestinian refugees and ongoing military incursions into Aida and Beit Jibrin camps,^31^ CHWs accomplished predetermined goals of at least 2 visits per month for every patient. Unlike other CHW programs that encountered low completion and follow-up rates,^43^ the unique setting of the refugee camp, in which social cohesion and community ties predominate, allowed for nearly immediate alliances between CHWs and their patients, resulting in zero refused visits by patients, and a 100% retention rate (30/30 diabetic patients).

Nearly immediate alliances formed between CHWs and their patients, resulting in zero refused visits and a 100% retention rate.

Immediate entry of in-visit measurements and lab tests into the KoboToolbox additionally allowed for continuous and frequent assessment of patient progress. This system expedited the recognition of patients with repeatedly elevated random blood glucose checks, prompting the H4P medical physician to accompany the CHWs on their visits to these patients and provide real-time care adjustments. Similarly, the centralization of patient information within 1 dataset accessible to CHWs facilitated frequent reviews of test results and measurement values by patients during CHW visits. This offered patients a sense of agency through data sovereignty^44^ and provided frequent reminders of their own diabetic progress or lack thereof, which may have been influential in motivating patients toward improved disease control.

These successes in program feasibility are reflected in the primary blood pressure and A1c results. A low exclusion cut-off for A1c (≤ 6.4%) may even underestimate the impact of the program given the lack of desirability of tighter control in patients with A1c less than 7% and in some patients with A1c of 7%–8%.^45^ Moreover, restriction of the study sample from which controls were selected to only those with a follow-up A1c (53.6% of the original population) suggests conservative results; any patient who failed to receive a second A1c within a year was automatically excluded from being selected as a control, resulting in a comparison of the CHW group to a group of control patients who were able to receive a follow-up A1c and thus were likely more motivated than potential controls who did not receive follow-up A1c testing.

Regarding the generalizability of the results indicating a positive association between CHW patients and improved diabetic status within 2 West Bank refugee camps, our cohort profile indicates similar baseline characteristics, or characteristics suggesting a higher-risk cohort, when compared to the general profile of diabetic patients living within West Bank refugee camps (Table 1). Per the UNRWA 2018 Annual Health Report, 34% of T2DM patients are treated with insulin as part of their management,^3^ similar to the 33% among patients receiving the intervention in this analysis. Regarding lifestyle behaviors, the patients in the CHW cohort reflected on average higher-risk behavior, with 26.7% self-reporting as smokers compared to 10.8% in the report, and 73.3% reporting physical inactivity, compared to 36.7% reported by UNRWA.

It is possible that the higher-risk behaviors found among the CHW study population compared to West Bank refugees generally are related to the populations’ proximity to a military base and their extremely high exposure to tear gas and home invasions. The challenges of implementing a CHW program in this setting may have positive implications in terms of expanding to camps subjected to less frequent military attacks. Alternatively, the conditions in Aida may have rendered them particularly vulnerable and with barriers to care that increased their likelihood of benefiting from such a CHW program.^46^ For example, preliminary data suggests support for the theory that a common cause for a patient's chronic disease transitioning from controlled to uncontrolled is related to psychosocial aspects of the occupation such as home invasions and the imprisonment of relatives.^47^ Similarly, residents within Aida and Beit Jibrin camps may have been better positioned to achieve positive results given their lack of proximity to a UNRWA clinic, raising questions as to the generalizability of these results to camps that do have more accessible UNRWA clinics.

While the work encompassed by this CHW intervention can be described in measurable terms, ranging from blood glucose checks to medication adherence support, the effects of this work may not be fully describable by its discrete elements. The therapeutic impact may rest in part on a sociopsychological intergenerational bolstering of the capacity to endure, manage, and resist hardships, including life under occupation as well as chronic diseases like hypertension and diabetes.^48^ Further study is warranted to better distinguish which parts of the CHW intervention, if they can be disentangled, are most effective in influencing improvements in disease control, particularly given the program’s resource-intensive character.

Further study is warranted to better distinguish which parts of the CHW intervention are most effective in improving disease control.

The H4P CHW program demonstrates that supplementation of patient-doctor interaction with paraprofessional services is both feasible and has potential for success within Palestine and possibly in other areas suffering from direct violence and extreme adversity. H4P is currently expanding to the largest refugee camp in the West Bank, Balata camp in the city of Nablus, where CHWs are working as of early 2022. Given the success and reasonable cost of this pilot initiative, scaling of the CHW model to other Palestinian refugee camps should be considered, particularly given there is substantial evidence that similar reductions in A1c result in cost savings and reductions in morbidity and mortality.^49^^–^^51^ Because UNRWA has a mandate that includes job creation in addition to health care and education for Palestinian refugees, this program is well suited to the institution’s scope and could be considered for incorporation into its noncommunicable disease program to enhance sustainability and institutionalization. However, a follow-up study with a robust analysis to calculate projected cost savings would be necessary if this were to be considered for a program still reliant on NGO funding.

Given the lack of randomization of patients, there may be other unmeasured and measured confounders, such as medication adherence or nutritional habits. Attempts to mitigate this included matching patients with controls, adjusting for potential confounders within the model, and assessing the nature by which patients were enrolled into the CHW program.

### Limitations

Other limitations include the inability to select controls by camp residence. Controls were selected from a medical database containing A1c results and demographic information on all adult diabetic patients receiving care at the UNRWA Bethlehem clinic, indicating refugee status, and signifying residence within Aida camp, Beit Jibrin camp, or Bethlehem Governorate. Since leaving a refugee camp often represents an advancement in socioeconomic status, this use of controls living within Bethlehem proper may indicate a higher level of socioeconomic status among the control group, which might be associated with improved diabetic care and greater medication affordability. Alternatively, living within the camps may be associated with high levels of social cohesion, which could also positively influence diabetes control. And because CHW patients were most commonly recruited into the program through snowballing, these patients may have had even stronger social connections than is typical in the camps.

## CONCLUSION

Despite their feasibility and potential for positive outcomes, community-led health initiatives are often overlooked and underfunded in settings of direct violence, including in Palestinian refugee camps. Yet, such programs can yield compounded benefits. In addition to providing employment opportunities, community-led health care initiatives actively tackle health inequities through efforts entirely rooted in and dependent on community members and staff, with little reliance on existing oppressive or external health structures. Improving health outcomes and chronic disease care through CHW programs and community-led initiatives is actionable, feasible, and promising in Palestinian refugee camps and other settings plagued by extreme adversity and geopolitical barriers to restitution.

## Supplementary Material

GHSP-D-22-00168-supplement.pdf

## Notes

**Peer Reviewed**

**First published online:** October 10, 2022.
