# Supplementary material for: Community Health Worker Program Outcomes for Diabetes and Hypertension Control in West Bank Refugee Camps: A Retrospective Matched Cohort Study
Source: Glob Health Sci Pract. 2022 Oct 31;10(5):e2200168. doi: 10.9745/GHSP-D-22-00168 (PMC9622278; doi:10.9745/GHSP-D-22-00168)
Supplement: GHSP-D-22-00168-supplement.pdf [file GHSP-D-22-00168-supplement.pdf]

Supplement to: Rimawi A, Shah A, Louis H, et al. Community health worker program outcomes in West Bank refugee camps: a retrospective matched cohort study. *Glob Health Sci Pract*. 2022;10(5):e2200168.
https://doi.org/10.9745/GHSP-D-22-00168

Supplement Table S1: Unverified A1c Values for Excluded Patients

Patient	Time since intervention (months)	Unverified A1c Values	Change in A1c
1	7	5.0	1.6
	13	6.6	
2	8	11.6	- 1.7
	14	9.9	
3	-1	7.5	0.5
	7	8	
4	-7	11.5	-3.1
	7	8.4	
5	11	8.1	-0.3
	17	7.8	

Supplement to: Rimawi A, Shah A, Louis H, et al. Community health worker program outcomes in West Bank refugee camps: a retrospective matched cohort study. *Glob Health Sci Pract*. 2022;10(5):e2200168. https://doi.org/10.9745/GHSP-D-22-00168

Supplement Table S2: Outcome Details

**Unadjusted key measures**			
**Predictor**	**Coefficient**	***P* Value**	**95% Confidence Intervals**
**Enrollment in CHW intervention**	-1.2	<.001	(-0.51, -1.88)
**No enrollment in CHW intervention**	0.08	.56	(-0.2, 0.36)
**Adjusted key measures from multivariable linear regression model**			
**Predictor**	**Coefficient**	***P* Value**	**95% Confidence Intervals**
**Enrollment in CHW intervention**	-1.4	<.001	(-0.66, -2.1)
**Intercept**	0.3	.71	(-1.5, 2.2)
**Hypertension**	-0.002	.99	(-0.59, 0.58)
**Smoking**	-0.64	.27	(-1.78, 0.5)
**Physical Activity**	0.07	.18	(-0.75, 0.9)
**Myocardial Infarction History**	-0.59	.08	(-1.2, 0.06)
**Stroke History**	0.82	.26	(-0.62, 2.3)

Shown are the mean values for the outcome in the CHW patient group and the non-CHW patient group. Calculation of the unadjusted mean A1c change was based on an indicator from an ordinary least-squares regression of the outcome, with no other covariates. Calculation of the adjusted A1c change was based on an indicator from an ordinary least-squares regression of the outcome with prespecified covariates. All confidence intervals (CIs) were calculated with the use of heteroskedasticity-robust standard errors. Prespecified covariates included history of myocardial infarction, history of stroke, physical activity status, smoking status, and hypertension status. All covariates were measured at time of baseline A1c and were extracted from the UNRWA Bethlehem clinic medical records.
